# SARS-CoV-2-positives Kind – Was tun bei unvermeidbarer inhalativer Narkoseeinleitung?

**DOI:** 10.1007/s00101-021-00941-8

**Published:** 2021-03-17

**Authors:** Nicolas Leister, Sirin Yücetepe, Christoph Ulrichs, Tobias Hannes, Uwe Trieschmann

**Affiliations:** Klinik für Anästhesiologie und Operative Intensivmedizin, Uniklinik Köln, Kerpener Straße 62, 50937 Köln, Deutschland

**Keywords:** COVID-19-Pandemie, Alternative Narkoseeinleitung, Schwieriger Venenzugang, Kinderanästhesie, Aerosol, COVID-19 pandemic, Alternative induction of anesthesia, Difficult venous access, Pediatric anesthesia, Aerosol

## Abstract

Die inhalative Anästhesieeinleitung hat bei Kindern aufgrund schwieriger Venenverhältnisse und insbesondere bei unkooperativen Patienten einen hohen Stellenwert. In der europaweiten Studie zu Komplikationen in der Kinderanästhesie (APRICOT-Studie) mit fast 30.000 eingeschlossenen Patienten wurde bei 48 % der Kinder die Narkose inhalativ eingeleitet.

Unter den Bedingungen der Coronapandemie stellt die inhalative Anästhesieeinleitung aufgrund der potenziellen Aerosolfreisetzung allerdings ein erhöhtes Infektionsrisiko dar. Für die Anästhesieeinleitung und die definitive Atemwegssicherung wird bei Erwachsenen und Kindern in der aktuellen Pandemiesituation eine „rapid sequence induction“ empfohlen.

Der vorliegende Fall demonstriert, dass es bei Kindern durchaus Situationen geben kann, in denen die inhalative Narkoseeinleitung unvermeidbar ist, und zeigt eine potenzielle Verfahrensweise zur Reduktion des Infektionsrisikos für das betreuende Anästhesiepersonal.

## Abstract (en)

Induction of anesthesia by inhalation is very common in children due to difficult venous conditions and especially in uncooperative children. During the study on complications in the pediatric anesthesia in Europe (APRICOT study) including almost 30,000 patients, 48% of the children were induced by inhalation.

Under the conditions of the corona pandemic, however, induction of anesthesia by inhalation represents an increased risk of infection due to the potential release of aerosols. Rapid sequence induction is recommended for anesthesia induction and definitive airway management for adults and children in the current pandemic situation.

The present case demonstrates that there can be situations in children in which induction of anesthesia by inhalation is unavoidable and shows a potential procedure for reducing the risk of infection for the anesthesia personnel.

## Einleitung

Kinder, die mit „severe acute respiratory syndrome coronavirus type 2“ (SARS-CoV-2) infiziert sind, haben häufig keine oder nur milde Symptome, trotzdem können sie das Virus übertragen und stellen eine potenzielle Infektionsquelle für ihre Umgebung dar [11].

Die Empfehlungen zum Umgang mit COVID-19-Erkrankten oder Patienten mit unbekanntem COVID-19-Status führen in der Anästhesiologie zu deutlichen Implikationen.

Die „rapid sequence induction“ (RSI) ist eine der wichtigen Empfehlungen. Wie aber soll bei Kindern vorgegangen werden, die einen schwierigen Venenstatus haben und die deshalb meistens inhalativ eingeleitet werden? Dazu erfolgt in dieser Kasuistik die Vorstellung und Diskussion des konkreten Falls eines SARS-CoV‑2 positiven Kindes.

## Fallbericht

Um die Mittagszeit wurde ein 14-Monate alter und 12 kg schwerer Junge mit Parotisschwellung von der Mutter in der pädiatrischen Notaufnahme vorgestellt. Das Kind wurde mit Verdacht auf einen Parotisabszess aufgenommen. Es erfolgten mehrere frustrane Venenpunktionsversuche in der Notaufnahme, um eine antibiotische Therapie zu beginnen. Das Kind und die Mutter (die vor 10 Tagen SARS-CoV-2-positiv getestet worden war) erhielten bei Aufnahme einen PCR-Test auf SARS-CoV‑2. Bis zum Vorliegen des Testergebnisses vergingen 6 h (Kind positiv auf SARS-CoV-2). Erst im Anschluss wurde der Patient bei der Anästhesie zur operativen Abszessspaltung angemeldet. Klinisch zeigte das Kind, abgesehen von subfebrilen Temperaturen, keine charakteristische COVID-19-Symptomatik.

## Klinischer Befund

Neben den organisatorischen hygienischen Vorsichtsmaßnahmen (persönliche Schutzausrüstung, Springer, adäquate Vorbereitung etc.) stellen sich anästhesiologisch einige besondere Fragen:

- Prämedikation, *ja* oder *nein*?
- Welche Einleitungsform? Gibt es Alternativen zur inhalativen Einleitung?
- Wie soll ein Zugang trotz schwieriger Venensituation etabliert werden?
- Atemwegssicherung mit Tubus oder mit Larynxmaske?
- Wie und wo soll das Kind nach der Beendigung des Eingriffs überwacht werden, um eine Verbreitung von Aerosolen zu vermeiden?

Aufgrund der Verzögerung der Anästhesievorstellung durch das Abwarten des Abstrichergebnisses wurde mit der Narkoseeinleitung erst gegen 19:00 Uhr begonnen, sodass das Kind bereits über 7 h nüchtern war. Durch die vorliegende Erkrankung, die lange Nüchternheit sowie die zahlreichen vorangegangenen Punktionsversuche zeigte sich das Kind bereits leicht somnolent, sodass von einer sedierenden Prämedikation abgesehen wurde. Da bei ansonsten gutem Ernährungszustand und nach der langen Nüchternheit keinerlei Venen sichtbar waren, entschied sich das Anästhesieteam für eine inhalative Anästhesieeinleitung, da auf diesem Wege die besten Erfolgsaussichten für eine erfolgreiche Venenpunktion erwartet wurden.

## Durchführung der Narkose

Damit die Narkoseeinleitung zügig erfolgen konnte, wurde das Kreissystem mit Sevofluran 8 % vorgefüllt. Unmittelbar nach Umlagerung des Kindes auf den OP-Tisch wurde die Maske dicht aufgesetzt. Dabei wurde der Frischgasfluss auf 1 l/min reduziert. Die Spontanatmung konnte in der Induktionsphase durchgehend erhalten werden. Nach erfolgreicher Venenpunktion im dritten Versuch erfolgte die Narkosevertiefung mit Remifentanil und Propofol. Testweise wurde eine Larynxmaske der 2. Generation eingesetzt, da man sich eine Reduktion der Atemwegsirritation und in der Folge geringere Hustenwahrscheinlichkeit versprach. Eine druckkontrollierte Beatmung mit einer Begrenzung des Spitzendrucks auf 14 mbar wurde begonnen. Es wurde lediglich ein PEEP von 2 mbar appliziert. Aufgrund der kurzen Operationsdauer (ca. 15 min), und da das Narkosebeatmungsgerät keine Leckage detektierte, was auch dem klinischen Eindruck entsprach, wurde die Larynxmaske belassen. Zur postoperativen Analgesie wurde Ibuprofen rektal verabreicht.

Die Operation erfolgte ohne jegliche Komplikationen; die Beatmung war immer suffizient und ohne Leckagezeichen. Zum Ende der Operationsmaßnahmen konnte der kleine Patient zügig in die Spontanatmung überführt sowie die Larynxmaske beim noch schlafenden Kind entfernt werden. Im weiteren Verlauf wurden dem Kind bei beginnender Unruhe 0,5 mg/kgKG Propofol injiziert und prophylaktisch 2 µg/kgKG Clonidin verabreicht. Zusätzlich bekam das Kind den Schnuller zurück. Die Mund-Nasen-Maske wurde wieder angelegt. Da diese für das Kind zu groß erschien, wurde sie leicht mit Pflasterstreifen fixiert. Anschließend erfolgte der problemlose Transport auf die pädiatrische Station, wo das Kind isoliert werden konnte.

## Diskussion

Ausgehend von diesem Fallbeispiel wollen wir einige grundsätzliche Überlegungen zur anästhesiologischen Versorgung von SARS-CoV-2-positiven Kindern darlegen und zur Diskussion stellen. Es gibt wenig Evidenz in diesem sensiblen Bereich: Ein Consensus Statement der Society for Pediatric Anesthesia’s Pediatric Difficult Intubation Collaborative und der Canadian Pediatric Anesthesia Society [12] fasst wichtige Aspekte zusammen, die wir im Folgenden berücksichtigen.

Anästhesisten sind aufgrund der Tätigkeit in der Nähe zu kontagiösem Aerosol und Sputum einer besonderen Gefahr ausgesetzt. Die nationalen und internationalen Empfehlungen zum Atemwegsmanagement bei COVID-19-positiven Patienten oder Patienten mit unbekanntem COVID-19-Status empfehlen aus diesem Grunde die i.v.-Einleitung im Sinne eine RSI, um die Aerosolproduktion und somit die Infektionsgefahr für das beteiligte medizinische Fachpersonal so gering wie möglich zu halten [4, 8].

Prinzipiell werden in der Kinderanästhesie ähnliche Vorgehensweisen empfohlen [12]. Da insbesondere in der Altersgruppe unter 2 Jahren die peripher venöse Erstpunktion lediglich bei der Hälfte der kleinen Patienten erfolgreich ist [5], müssen auch andere Wege der Anästhesieeinleitung erwogen werden [7, 14].

Die Voraussetzung zur Behandlung COVID-19-positiver oder COVID-19-Status-unbekannter pädiatrischer Patienten ist das Vorhandensein suffizienter persönlicher Schutzausrüstung. Damit kann die Infektionsübertragung vom Patienten auf das medizinische Fachpersonal deutlich reduziert werden [15]. Im vorliegenden Fall bestand die Schutzausrüstung des anästhesiologischen Teams aus OP-Haube, „face shield“, FFP-3-Maske, Schutzkittel sowie medizinischen Handschuhen.

Die Nutzung von Schleusen und Niederdruckoperationsräumen wird empfohlen [8]. Auch die Nutzung von Videolaryngoskopen scheint einen Stellenwert zur Reduktion der COVID-19-Transmission zu haben [8].

Obwohl es einen gewissen Trend zur Dosisreduktion der medikamentösen Prämedikation gibt, so ist bei SARS-CoV-2-positiven Kindern eine adäquate Prämedikation (Midazolam, ggf. zusätzlich Ketamin oral) unerlässlich, um ein ruhiges Kind und damit eine höhere Sicherheit des agierenden Personals zu erreichen [10].

Eine Einleitung mittels nasaler Applikation der Medikation ist aufgrund der potenziellen Aerosolkonfrontation für den Durchführenden als gefährdend und somit nicht akzeptabel zu betrachten. Eine rektale Anästhesieeinleitung ist zeitlich und auch im Hinblick auf die Wirkung ungewiss, stellt aber bei ausreichender Erfahrung mit dem Verfahren ggf. eine Option dar. Intramuskuläre Einleitungsversuche sind obsolet, da sie Schmerzen verursachen und ein schreiendes Kind unbedingt vermieden werden muss.

Die Nutzung des Ultraschalls zur peripheren Venenpunktion nach vorheriger EMLA-Applikation darf nicht unerwähnt bleiben und sollte regelmäßig geübt werden, allerdings ist hierzu ein kooperativer Patient (z. B. nach suffizienter Prämedikation) notwendig. Die Etablierung eines intraossären Zugangsweges ist natürlich jederzeit möglich, sollte jedoch nach Risiko-Nutzen-Abwägung dem vitalen Notfallgeschehen vorbehalten bleiben.

Daraus resultierend bleibt in einigen wenigen Fällen lediglich die inhalative Narkoseinduktion mit allen Implikationen: Jeder in der Kinderanästhesie tätige kennt den charakteristischen Geruch von Sevofluran im Einleitungsraum. Als ein Anzeichen von Aerosolverbreitung ist dies allerdings in den Zeiten der COVID-19-Pandemie als besonders kritisch anzusehen. Es ist davon auszugehen, dass hierbei selbst bei kooperativen Kindern eine beträchtliche Menge an infektiösen Aerosolen entstehen kann. Allerdings gibt es Hinweise, dass die Maskenbeatmung eine niedrigere Infektionsgefahr für den Durchführenden darstellt als die Intubation [18].

Um das Risiko für das medizinische Personal zu minimieren, bedarf es einer umsichtigen Handlungsweise sowie spezieller organisatorischer Maßnahmen:

- Die Anästhesieeinleitung sollte möglichst nur durch einen im Atemwegsmanagement sehr *erfahrenen Kinderanästhesisten* und eine entsprechend erfahrene Anästhesiepflegekraft durchgeführt werden. Vor Beginn der Einleitung sollte das geplante Vorgehen mit allen Beteiligten eingehend besprochen werden [10].
- Die *persönliche Schutzausrüstung* sollte vollständig und korrekt angelegt sein (dies muss gegenseitig überprüft werden!). Es sollten ausschließlich für den Prozess wirklich notwendige Personen anwesend sein.
- Ein *weiterer erfahrener Anästhesist* in kompletter Schutzausrüstung *sowie ein pflegerischer Springer ***vor** dem Anästhesieeinleitungsraum sind unabdingbar, um im Bedarfsfall unterstützend tätig zu werden.

Für das weitere Vorgehen besteht die Kernaufgabe darin, alle Maßnahmen zu ergreifen, die eine Aerosolfreisetzung verhindern bzw. minimieren:

- Um größtmögliche Patienten-Compliance zu erreichen, sollte unbedingt eine *medikamentöse Prämedikation* erfolgen [10]. Auf diesem Wege ist in den meisten Fällen ein dichtes Aufsetzen der Beatmungsmaske möglich, sodass die Aerosolfreisetzung weitestgehend reduziert wird.
- Vor Beginn der eigentlichen Narkoseinduktion sollte das *Narkosesystem bei unruhigen Kindern bereits mit Sevofluran „geflutet“* sein, damit die inhalative Einleitung zügig erfolgen kann. Bei *kooperativen Patienten kann durch die langsame Steigerung der inspiratorischen Narkosegaskonzentration* die Compliance gefördert bzw. erhalten werden.
- Die Beatmungsdrücke sollten so niedrig wie möglich gehalten werden; deshalb sollte die *Spontanatmung erhalten* bleiben, CPAP zum Offenhalten des Atemweges kann appliziert werden.
- Falls auf eine Druckunterstützung nicht verzichtet werden kann, sollte der Beatmungsdruck auf 10–14 mbar limitiert werden; dies gelingt oftmals besser mit einer maschinellen als mit einer manuellen Beatmung.
- Bei dichtsitzender Maske ist eine *Minimierung des Frischgas-Flow* auf bis zu 1 l/min möglich [17], was die Aerosolfreisetzung reduziert (mit steigendem Flow steigt auch die Aerosolproduktion [3, 6]).
- *Vor (!) dem Absetzen der Maske ist der Gasfluss zu beenden und das Y‑Stück zu verschließen *(z. B. mit einer passenden (roten) Verschlusskappe), um die Aerosolverteilung im Raum zu verhindern. Hierbei ist die resultierende Kontamination des Verschlussmittels zu beachten.
- Um eine unkontrollierte Aerosolfreisetzung durch Husten nach Entfernung von Larynxmaske oder Tubus zu verhindern, sind eine *adäquate Analgesie und Sedierung* notwendig.
- Mund und Nase des Kindes sollten schnellstmöglich mit einer *Mund-Nasen-Maske* bedeckt werden.

Ein Abdecken des Patientengesichtes (Box oder Folie) zur Reduktion der Aerosolbewegung im Raum wurde zwar zu Beginn der Pandemie empfohlen [12], resultiert allerdings eher in einer Gefährdung des Patienten sowie des medizinischen Personals. Deshalb warnt die FDA inzwischen vor einem solchen Vorgehen [1, 2, 16].

Das Anlegen einer medizinischen Mund-Nasen-Maske konnte in dem vorliegenden Fall erfolgreich umgesetzt werden. Die Akzeptanz bei kleinen Kindern (<2 Jahre) ist zumeist nicht gegeben [11]; ein Versuch der korrekten Platzierung einer Mund-Nasen-Maske unter Zwang führt durch Agitation u. U. zu vermehrter Aerosolfreisetzung und ist somit nicht zielführend.

Eine weitere Möglichkeit zur Reduktion der Aerosolfreisetzung könnte die Doppelmaske (Fa. Medicvent AB, Umea, Schweden) sein, welche in manchen kinderanästhesiologischen Abteilungen zur inhalativen Einleitung genutzt wird und aktiv um die Maske entstehende Aerosole absaugt [9]. Allerdings gibt es keine Studien in Hinsicht auf Reduktion der Virusfreisetzung, und die hygienische Aufbereitung nach Nutzung ist fraglich.

Im vorliegenden Fall bleibt zu diskutieren, ob die präoperativ frustranen Punktionsversuche und die daraus resultierende Verzögerung in der Initialisierung der antibiotischen Therapie die Anlage eines intraossären Zugangs begründen. Dieser hätte in der Folge auch zur Narkoseeinleitung im Sinne einer RSI genutzt werden können. Allerdings ist die semielektive Nutzung der intraossären Infusion in dieser Klinik bisher nicht etabliert. Das Anästhesieteam hatte ein automatisiertes intraossäres Zugangssystem zur Verfügung und einen etwaigen Einsatz bei Komplikationen oder wiederholter peripherer Fehlpunktion abgesprochen [13].

Zusammenfassend bleibt zu sagen, dass unseres Erachtens bei gegebener Indikation eine inhalative Einleitung durch ein erfahrenes kinderanästhesiologisches Team auch in Zeiten der COVID-19-Pandemie durchgeführt werden kann. Zur Erhöhung der Sicherheit für das anästhesiologische Personal sollten die oben genannten Voraussetzungen erfüllt werden. Absolut unabdingbar ist die sichere Anwendung der persönlichen Schutzausrüstung.

## Fazit

Eine inhalative Narkoseeinleitung ist auch in der COVID-19-Pandemie möglich, wenn:

- persönliche und infrastrukturelle Hygienemaßnahmen eingehalten werden,
- die Expertise im durchführenden Team vorliegt,
- Patienten kooperativ sind (medikamentöse Prämedikation!),
- Spontanatmung lange erhalten bleibt,
- der Gasfluss minimiert wird,
- das Beatmungssystem (inkl. Patient) ohne Leckage ist,
- Beatmungsdiskonnexionen vermieden werden.
